# Factors associated with exclusive breastfeeding practices among mothers in dubti town, afar regional state, northeast Ethiopia: a community based cross-sectional study

**DOI:** 10.1186/s13006-016-0064-y

**Published:** 2016-03-18

**Authors:** Misgan Legesse Liben, Yohannes Bacha Gemechu, Mulugeta Adugnew, Adugnaw Asrade, Belete Adamie, Ehitemelak Gebremedin, Yibel Melak

**Affiliations:** Department of Public Health, College of Medical and Health Sciences, Samara University, Afar, Ethiopia; Department of Nursing, College of Medical and Health Sciences, Samara University, Afar, Ethiopia; Ministry of Health, Addis Ababa, Ethiopia

**Keywords:** Exclusive breastfeeding, Dubti, Town, Afar, Northeast Ethiopia

## Abstract

**Background:**

Exclusive breastfeeding for the first 6 months of life is recommended internationally. This study aimed to investigate exclusive breastfeeding practices and associated factors among mothers of infants aged less than 6 months.

**Methods:**

A community based cross-sectional study was conducted on mother-infant pairs in Dubti town in May, 2015. In this study, exclusive breastfeeding was defined as an infant’s breast milk consumption without supplementation of any type of food or drink, except for vitamins, minerals and necessary medications in the 24 h preceding the survey. Descriptive statistics, bivariate and multivariable logistic regression analysis were employed to identify the factors associated with exclusive breastfeeding practices. Variables with a *p*-value < 0.05 in the multivariable model were identified as predictors of exclusive breastfeeding practices.

**Results:**

Exclusive breastfeeding under 6 months was practiced by 81.1 % (95 % Confidence Interval [CI] 77.0, 85.0 %) of mothers of infants aged less than 6 months. The median duration of exclusive breastfeeding for infants less than 6 months was 3 months. Multivariable logistic regression analysis showed that initiation of breastfeeding within 1 h after birth (Adjusted Odds Ratio [AOR] 5.46; 95 % CI 1.93, 15.41), age of infants of less than 2 months (AOR 7.03; 95 % CI 2.16, 22.88), being a housewife (AOR 4.81; 95 % CI 2.30, 10.06) and mothers who received postnatal counseling (AOR 3.88; 95 % CI 1.88, 7.99) were positive predictors of exclusive breastfeeding.

**Conclusion:**

The study revealed that exclusive breastfeeding under 6 months using 24-h recall method was lower than the World Health Organization recommendation. Therefore, interventions could focus on educating mothers the importance of timely initiation of breastfeeding and postnatal care in the study area.

## Background

Breastfeeding provides the ideal food for the healthy growth and development of infants. It is also an integral part of the reproductive process with important implications for maternal health. It has an important contraceptive effect in the first 6 months after delivery. Breast milk is the natural first food for infants, which provides all the energy and nutrients that the infant needs for the first 6 months of life. It promotes sensory and cognitive development, and protects the infant against infectious and chronic diseases [1].

Exclusive breastfeeding is an international and national recommendation for children up to 6 months of age, defined as an infant’s breast milk consumption without supplementation of any type of food or drink, except for vitamins, minerals and necessary medications up to the age of 6 months [2, 3]. Exclusive breastfeeding provides all the nutrients and water that a baby needs to grow and develop in the first 6 months [4, 5]. Therefore, to enable mothers to establish and sustain exclusive breastfeeding for the first 6 months, WHO recommends mothers to initiate breastfeeding early, exclusively breastfed, breastfeed on demand, and not to use bottles, teats or pacifiers [1].

Suboptimal breastfeeding results in an increased risk of child morbidity and mortality in the first 2 years of life. It results in more than 800,000 child deaths annually worldwide [6]. Exclusive breastfeeding provides both nutrition and protection from illnesses. If every infant is exclusively breastfed from birth for 6 months, an estimated 1.3 million lives will be saved worldwide every year [1]. A cross-sectional survey in Vietnam in 2011 stated that infants who had breastfed exclusively to 6 months were less likely to suffer from acute respiratory tract infection and diarrhea compared to non-exclusively breastfed infants [7].

Breastfeeding is almost universal, about 98 % of all children ever breastfed, in Ethiopia and the recent Demographic and Health Survey of Ethiopia estimated that only 52 % of infants under 6 months of age benefited from exclusive breastfeeding [5]. Despite this fact, there are limited studies on exclusive breastfeeding practices in pastoralist communities of Ethiopia. Therefore, it is vital to assess the factors affecting exclusive breastfeeding in Afar Regional State. The findings of this study will be vital for health service providers, policy makers and program managers to design intervention strategies that may promote optimal breastfeeding practices in Afar Regional State and the study area.

## Methods

### Study setting and participants

This study was conducted in Dubti town in May, 2015. Dubti town is found in zone one of Afar regional state of Ethiopia. It is located 570 kms and 12 kms from Addis Ababa and Samara, respectively. Dubti district is bordered on the south by Somali Regional state, on the Southwest by Mille, on the west by Chifra, on the Northwest by the administrative zone 4, on the north by Kori, on the Northeast by Elidar, on the east by Aysaita and on the Southeast by Afambo districts.

A quantitative community based cross sectional study was employed on mothers of infants aged less than 6 months. A sample size of 346 was calculated using a single population proportion formula;

$$ \mathrm{n}=\frac{{\left(\mathrm{z}\frac{\upalpha}{2}\right)}^2\;\mathrm{p}\;\left(1\;\hbox{-}\;\mathrm{p}\right)}{{\mathrm{d}}^2} $$

Where *n* = required sample size, $z\;\frac{\alpha }{2}$ = critical value for normal distribution at 95 % confidence level (1.96), *p* = prevalence of exclusive breastfeeding in Goba district of Ethiopia (71.3 %) [8], d = 0.05 (5 % margin of error) and an estimated non-response rate of 10 %.

### Sampling procedure and data collection process

A pre-survey was done before the actual period of data collection to identify the households which have the targeted mother-child pairs. At the time of survey, from each household unit one eligible mother of infants aged less than 6 months was selected using systematic random sampling technique after identifying an initial starting household by lottery. If there were more than one mother with infants aged less than 6 months in one household unit, one mother with the youngest infant was selected. If mothers had twin infants aged less than 6 months, one infant was selected by lottery. At the time of data collection mothers who were seriously ill and unable to communicate were excluded from the study.

Data were collected by five BSc nursing students using a pre-tested, structured and interviewer administered questionnaire. In this study exclusive of breastfeeding was assessed by using 24-h recall method. The questionnaire was prepared first in English and translated into Qafaraf (the local language), and then back into English to check its consistency. The final Qafaraf (local language) version of the questionnaire was used to collect the data.

### Study variables

In this study, the dependent variable was exclusive breastfeeding practice among mothers of infants aged less than 6 months. Exclusive breastfeeding was defined as an infant’s breast milk consumption without supplementation of any type of food or drink, except for vitamins, minerals and necessary medications in the last 24 h preceding the survey [9]. The independent variables were maternal characteristics (age, educational status, religion, occupation, marital status, parity and ethnicity), household characteristics (household head, and family size), infant characteristics (sex, age), husband educational status, health service related variables (site of delivery, mode of delivery, antenatal care and postnatal care). In addition, infant feeding counseling during antenatal care and postnatal care was reported by the mothers. Early initiation of breastfeeding was defined as breastfeeding initiation within 1 h after birth.

### Data quality control

BSc nursing students were recruited as data collectors. The data collectors were trained for 2 days on the study instrument, consent form, how to interview and data collection procedures. The questionnaire was pretested, and then the pretest amendments were made accordingly. The supervisors (two health professionals having a master’s degree) had checked the day to day activity of data collectors regarding the completion of questionnaires, clarity of responses and proper coding of the responses.

### Data processing and analysis

The data were checked for completeness and inconsistencies. It was also cleaned, coded and entered on to EpiData version 3.02, then exported to SPSS 20.0 statistical package for analysis.

Univariable logistic regression analysis was performed. The crude odds ratio (COR) with 95 % confidence interval was estimated to assess the association between each independent variable and the outcome variable. Variables with *p*-value < 0.05 in the univariable logistic regression analysis were considered in the multivariable logistic analysis.

The Hosmer-Lemeshow goodness-of-fit with enter procedure was used to test for model fitness. Adjusted Odds Ratio (AOR) with 95 % confidence interval was estimated to assess the strength of the association, and a *p*-value < 0.05 was used to declare the statistical significance in the multivariable analysis. Variables with *p*-value < 0.05 in the multivariable logistic regression analysis were considered as significant and independent predictors of exclusive breastfeeding.

### Ethical consideration

The study was approved by Samara University, Department of Nursing (no approval numbers are given). An official letter was written from nursing department to Dubti town administration office. The participants enrolled in the study were informed about the study objectives, expected outcomes, benefits and the risks associated with it. A written consent was taken from the participants before the interview. Confidentiality of responses was maintained throughout the study.

## Results

### Socio-demographic characteristics of study participants

A total of 333 mother-infant pairs were included in the study, making a response rate of 96.2 %. Nearly half of the mothers were Afar in Ethnicity. About 73 % of the respondents were Muslims,. Moreover 82 % of mothers were in the age group of 20–34 years, and 24.3 % were household heads (Table 1).

**Table 1:** Socio-demographic characteristics of mother-infant pairs in Dubti town, Northeast Ethiopia, 2015

Variable	Frequency	Percent (%)
Age of mother (in year) (*n* = 333)		
<20	14	4.2
20–34	273	82.0
>34	46	13.8
Mean (± SD) age of mother	27.02 (±5.45) years	
Ethnicity (*n* = 333)		
Afar	168	50.5
Amhara	139	41.7
Tigray	26	7.8
Maternal religion (*n* = 333)		
Muslim	256	76.9
Orthodox	71	21.3
Protestant	6	1.8
Maternal occupation (*n* = 333)		
Housewife	280	84.1
Government employed	42	12.6
Others^a^	11	3.3
Maternal education status (*n* = 333)		
No formal education	165	49.5
Formal education	168	50.5
Maternal marital status (*n* = 333)		
Currently unmarried^b^	14	4.2
Married	319	95.8
Husband educational status (*n* = 319)		
No formal education	80	25.1
Formal education	229	74.9
Household head (*n* = 333)		
Mothers of the index infant	81	24.3
Fathers of the index infant	252	75.7
Sex of the index infant (*n* = 333)		
Male	203	61.0
Female	130	39.0
Age of the index infant (in month) (*n* = 333)		
<2	70	21.0
2–3	165	49.6
4–6	98	29.4
Mean (± SD) age of infants	3.55 (±1.24) months	
Family size (*n* = 333)		
2	6	1.8
3–4	160	48.0
≥5	167	50.2
Parity of respondent (*n* = 333)		
1	82	24.6
2–4	180	54.1
≥5	71	21.3

^a^farmer, daily laborer, trader

^b^single, divorced, widowed

### Maternal health service related and obstetric variables

Three hundred twenty (96.1 %) of mothers had attended antenatal care visit, 302 (90.7 %) gave birth at health institution and 212 (63.7 %) attended postnatal care visit. Of those who had attended antenatal care visits 58.4 % were counseled on infant feeding practices (Table 2).

**Table 2:** Distribution of mothers based on health service related and obstetrics in Dubti town, Northeast Ethiopia, 2015

Maternal health services	Frequency	Percent (%)
Antenatal care (ANC) (*n* = 333)^a^		
Yes	320	96.1
No	13	3.9
Infant feeding counseling during ANC (*n* = 320)		
Yes	187	58.4
No	133	41.6
Place of birth (*n* = 333)		
Health institution	302	90.7
Home	31	9.3
Mode of delivery (*n* = 333)		
Vaginal delivery	313	94.0
Caesarean section delivery	20	6.0
Postnatal care (PNC) (*n* = 333)^a^		
Yes	212	63.7
No	121	36.3
Infant feeding counseling during PNC (*n* = 212)		
Yes	162	76.4
No	50	23.6

^a^at least one visit

### Breastfeeding patterns

All mothers surveyed in Dubti town had ever breastfed their index infant. About 93 % of mothers had initiated breastfeeding within 1 h of birth. Exclusive breastfeeding under 6 months was practiced by 81.1 % (95 % CI 77.0, 85.0 %) of mothers of infants aged less than 6 months. The median duration of exclusive breastfeeding for infants less than 6 months was 3 months. Moreover, prelacteal feeding and colostrum avoidance were practiced by 16.8 and 15.6 % of mothers of infants aged less than 6 months, respectively (Table 3).

**Table 3:** Infant feeding practices among mothers of infants aged less than 6 months in Dubti town, Northeast Ethiopia, 2015

Variables	Frequency (n)	Percent (%)
Early breastfeeding (*n* = 333)		
Yes	309	92.8
No	24	7.2
Prelacteal feeding (*n* = 333)		
Yes	56	16.8
No	277	83.2
Colostrum feeding (*n* = 333)		
Yes	281	84.4
No	52	15.6
Mothers believe that EBF is enough for the first six months (*n* = 333)		
Yes	273	82.0
No	60	18.0
EBF practice under six months		
Yes	270	81.1
No	63	18.9

*Abbreviations*: *EBF* exclusive breastfeeding

### Factors associated with exclusive breastfeeding practices

The univariable logistic regression analysis showed that early initiation of breastfeeding, mothers who know that exclusive breastfeeding is enough for the first 6 months, colostrum feeding, age of infants of less than 2 months, maternal occupation and mother who got infant feeding counseling during postnatal care visit were associated with exclusive breastfeeding (Table 4).

**Table 4:** Crude and adjusted odds ratios of the factors associated with exclusive breastfeeding among mothers of infants aged less than 6 months in Dubti town, 2015

Variable	EBF *n* (%)	COR (95 % CI)	AOR (95 % CI)
Early breastfeeding initiation			
Yes	257 (83.2)	4.18 (1.78, 9.85)*	5.46 (1.93, 15.41)*
No	13(54.2)	1	1
EBF is enough for the first six months			
Yes	232 (85.0)	3.28 (1.76, 6.10)**	1.76 (0.85, 3.65)
No	38 (63.3)	1	1
Colostrum feeding			
No	33 (63.5)	1	1
Yes	237 (84.3)	3.10 (1.62, 5.94)*	2.03 (0.95, 4.32)
Age of the index infant (in month)			
<2	66 (94.3)	1.58 (0.88, 2.85)*	7.03 (2.16, 22.88)*
2–3	133 (80.6)	6.28 (2.08, 18.89)	1.56 (0.79, 3.05)
4–6	71 (72.4)	1	1
Maternal occupation			
Housewife	238 (85.3)	3.99 (2.11, 7.54)**	4.81 (2.30, 10.06)**
Other^a^	32 (59.3)	1	1
Infant feeding counseling during PNC visit			
No	145 (89.5)	3.14 (1.71, 5.75)**	3.88 (1.88, 7.99)**
Yes	125 (73.1)	1	1

^a^farmer, daily laborer, trader, government employed

*Abbreviations*: *PNC* postnatal care, *EBF* exclusive breastfeeding, *COR* crude odds ratio, *AOR* adjusted odds ratio, *CI* confidence interval

*significant at *p* < 0.05

**significant at *p* < 0.001

Multivariable logistic regression analysis showed that early initiation of breastfeeding, age of infants of less than 2 months, maternal occupation and mothers who got infant feeding counseling during postnatal care visit remained significant as independent positive predictors of exclusive breastfeeding. Mothers who initiated breastfeeding early were more likely to fed their infants exclusively compared to mothers who initiated breastfeeding later (Adjusted Odds Ratio [AOR] 5.46; 95 % Confidence Interval [CI] 1.93, 15.41). Compared with infants in the age range of 4 to 6 months, infants less than 2 months were seven times more likely to be breastfed exclusively (AOR 7.03; 95 % CI 2.16, 22.88). Compared to employed mothers, housewife (unemployed) mothers were more likely to practice exclusive breastfeeding (AOR 4.81; 95 % CI 2.30, 10.06). Mothers who received infant feeding counseling during postnatal care visit were nearly four times (AOR 3.88; 95 % CI 1.88, 7.99) more likely to breastfed their infants exclusively compared to those who did not get counseling during postnatal care (Table 4).

## Discussion

This study aimed to determine the prevalence of exclusive breastfeeding and associated factors in Dubti town. The prevalence of exclusive breastfeeding (EBF) under 6 months was 81.1 %, which is almost similar to the Ambo district prevalence of EBF [10]. But this finding is higher than the prevalence of EBF in Debre Markos town (60.8 %) [11], Gozamen district (61.3 %) [12], Mecha district (47.13 %) [13], Goba district (71.3 %) [8], Gondar town (52.5 %) [14] and Kenya (45.5 %) [15]. However, the prevalence of exclusive breastfeeding is lower than WHO recommendation of 90 % [16, 17].

The multivariable logistic model showed that age of infants was a predictor of exclusive breastfeeding practice. Compared with infants in the age group of 4 to 6 months, the infants aged less than 2 months were seven times more likely to be exclusively breastfed. Infants in the age group 2–3 months were 1.6 times more likely to be exclusively breastfed when compared to those infants in the age group 4–6 months. This shows that as the age of the infant approached 6 months, the rate of exclusive breastfeeding decreased significantly. This is similar to other studies [8, 15, 18, 19]. This might be due to the fact that postpartum care is given in the first few months after delivery where mothers stay at home, creating the opportunity to breastfeed their infant exclusively.

This study revealed that mothers who initiated breastfeeding early were more likely to practice exclusive breastfeeding compared to mothers who initiated breastfeeding later. In Mecha district mothers who initiated breastfeeding immediately after birth were more likely to practice exclusive breastfeeding than those who did not initiate breastfeeding immediately after birth [13]. This may due to the fact that late initiation of breastfeeding is associated with decreased newborn-mother bonding and then inadequate maternal breast milk secretion. This in turn may lead to early introduction of other foods and/or drinks.

In Dubti town being a housewife shows positive association with exclusive breastfeeding practices compared to employed mothers. Similar findings were obtained at Goba district [8], Ambo district [10], Dabat district [20], Bahir Dar city [19], Gondar [13] and Debre Markos towns [11, 12]. This could be due to the fact that housewives (unemployed mothers) have more time to be with their infant throughout the whole day and can provide breastfeeding on demand. On the other hand employed mothers may not have frequent contact with their infant. This hinders proper breastfeeding practices, and exclusive breastfeeding.

Mothers who had received infant feeding counseling during the postnatal visit were more likely to breastfed their infants exclusively. This is in line with a study from Mecha district where mothers who received PNC counseling on infant feeding were more likely to practice EBF compared to those who received no counseling during postnatal care [13] and also similar to findings at Debre Markos [11], and Addis Ababa [21]. This shows that postnatal period is an appropriate time to provide important infant feeding messages.

The study could have the following limitations. Firstly, it was difficult to establish a cause-effect relationship. Secondly, the information obtained from mothers having children aged less than 24 months might be subject to recall bias. Finally, using the previous 24 h recall period will cause the proportion of exclusively breastfed infants to be overestimated, as some infants who are given other liquids irregularly may not have received them in the day before the survey. However, due attention was given to the study procedures, including the process of training, and close supervision throughout the field activities. The study also shares the limitation of cross sectional study design.

## Conclusion

The study revealed that EBF of infants under 6 months using the 24-h recall method was about 80 % in Dubti town. Initiating breastfeeding within 1 h after birth, infants aged less than 2 months, unemployed (housewife) mothers and infant feeding counseling during postnatal care were positive predictors of exclusive breastfeeding. Therefore, creating a breastfeeding enabling working environment to include breastfeeding breaks and paid maternity leave, and educating mothers the importance of early initiation of breastfeeding and postnatal care visit will improve exclusive breastfeeding practices in the study area.
